# Antidepressants in epilepsy

**DOI:** 10.1192/j.eurpsy.2022.761

**Published:** 2022-09-01

**Authors:** M.M. Gutiérrez Rodríguez, M.D.L.A. Corral Y Alonso, C. Moreno Menguiano, F. Garcia Sánchez, J.J. Vazquez Vazquez, S.M. Bañón González

**Affiliations:** 1 HOSPITAL UNIVERSITARIO DE MÓSTOLES, Psychiatry, MÓSTOLES (MADRID), Spain; 2 Centro de Salud Mental de Móstoles / Hospital Universitario de Móstoles, Psychiatry, Móstoles, Spain; 3 Hospital Infanta Sofía, Psiquiatría, Madrid, Spain

**Keywords:** Antidepressants, Epilepsy, Depression, Pharmacotherapy

## Abstract

**Introduction:**

Depressive disorders are one of the most frequent psychiatric comorbidity in epilepsy and they have a negative impact on the quality of life. Depression often requires antidepressant treatment. However, it is often left untreated in people with epilepsy, in part due to fear that antidepressants could cause seizures.

**Objectives:**

The goal of this study was to do a review and describe the evidence of the efficacy and safety of pharmacological treatment for depression in epilepsy.

**Methods:**

Review of literature sources were obtained through electronic search in PubMed database with special focus in papers published in the last 5 years.

**Results:**

The existing evidence of the effectiveness of antidepressants in treating depressive symptoms associated with epilepsy is still limited and response rate was highly variable. It is essential first to optimize seizure control and minimize unwanted antiepileptic drug-related side effects. As the first line of treatment you should consider the use of SSRI or IRSN. The improvement in depressive symptoms ranged from 25% to 82% according to the different studies and depending on the antidepressant administered. A review of the literature indicates that the risk of antidepressant-associated seizures is low although some antidepressants such as amoxapine or bupropion are not recommended.

**Conclusions:**

There are few comparative data to support the choice of antidepressant drug or drug class in terms of efficacy or safety for the treatment of people with epilepsy and depression. It would be important to design controlled trials of antidepressants in large cohorts of participants with epilepsy and clinically significant depression.

**Disclosure:**

No significant relationships.

